# Effects of Gamma and Electron Radiation on the Structural Integrity of Organic Molecules and Macromolecular Biomarkers Measured by Microarray Immunoassays and Their Astrobiological Implications

**DOI:** 10.1089/ast.2016.1645

**Published:** 2018-11-29

**Authors:** Yolanda Blanco, Graciela de Diego-Castilla, Daniel Viúdez-Moreiras, Erika Cavalcante-Silva, José Antonio Rodríguez-Manfredi, Alfonso F. Davila, Christopher P. McKay, Victor Parro

**Affiliations:** ^1^ Department of Molecular Evolution, Centro de Astrobiología (INTA-CSIC), Torrejón de Ardoz, Madrid, Spain.; ^2^ Space Science Division, NASA Ames Research Center, Moffett Field, California, USA.

**Keywords:** Electron radiation, Gamma radiation, Molecular biomarker, Antibody microarray, Epitope, Immunoidentification, Planetary exploration

## Abstract

High-energy ionizing radiation in the form of solar energetic particles and galactic cosmic rays is pervasive on the surface of planetary bodies with thin atmospheres or in space facilities for humans, and it may seriously affect the chemistry and the structure of organic and biological material. We used fluorescent microarray immunoassays to assess how different doses of electron and gamma radiations affect the stability of target compounds such as biological polymers and small molecules (haptens) conjugated to large proteins. The radiation effect was monitored by measuring the loss in the immunoidentification of the target due to an impaired ability of the antibodies for binding their corresponding irradiated and damaged epitopes (the part of the target molecule to which antibodies bind). Exposure to electron radiation alone was more damaging at low doses (1 kGy) than exposure to gamma radiation alone, but this effect was reversed at the highest radiation dose (500 kGy). Differences in the dose–effect immunoidentification patterns suggested that the amount (dose) and not the type of radiation was the main factor for the cumulative damage on the majority of the assayed molecules. Molecules irradiated with both types of radiation showed a response similar to that of the individual treatments at increasing radiation doses, although the pattern obtained with electrons only was the most similar. The calculated radiolysis constant did not show a unique pattern; it rather suggested a different behavior perhaps associated with the unique structure of each molecule. Although not strictly comparable with extraterrestrial conditions because the irradiations were performed under air and at room temperature, our results may contribute to understanding the effects of ionizing radiation on complex molecules and the search for biomarkers through bioaffinity-based systems in planetary exploration.

## 1. Introduction

The study of the effects of ionizing radiation on the organic molecules with abiotic or biological origin or even whole living beings is of major relevance in many research areas, especially in planetary exploration. Planetary surfaces may be exposed to high radiation doses due to a lack of an appreciable atmospheric or magnetic field, which critically affect their habitability (Dartnell, 2011). There is now compelling evidence of past habitable conditions on the surface of Mars (Leshin *et al.*, 2013), and of the existence of oceans of liquid water beneath the ice shell of Europa and Enceladus (Carr *et al.*, 1998; Waite *et al.*, 2009; Spencer and Nimmo, 2013). In the case of Enceladus, direct measurements of ocean materials have revealed the presence of organic compounds (Postberg *et al.*, 2009, 2011; Waite *et al.*, 2009). These findings motivate the search for evidence of life *in situ* with robotic missions, or in the future when it becomes economically and technically feasible, in samples returned to Earth.

The chemical and structural versatility of the biological polymers and the specificity of certain molecules synthesized by living organisms are perhaps one of the most direct and unambiguous signs of extant or extinct life (Pace, 2001; McKay, 2004; Summons *et al.*, 2008; Lunine, 2009; Davila and McKay, 2014). Indeed, most theories on the origin of biological organization assume that the detection of organic molecules analogous to nucleic acids or peptides with length in the range of tenths of monomers in a sample would be difficult to refute as a successful life detection experiment (Szostak and Ellington, 1993). However, biogenic organic molecules are susceptible to chemical and physical degradation after organisms die, and this constrains their preservation potential in the geological record (Eigenbrode, 2008). This could be a major limiting factor in the search for evidence of life on planetary environments where any organic biosignatures near the surface might have been exposed to physical and chemical degradation produced by environmental factors, for example, ultraviolet and ionizing radiation or powerful oxidants such as perchlorates for timescales of millions to billions of years.

One of the most pervasive long-term agents of organic molecule degradation on planetary surfaces is ionizing radiation in the form of galactic cosmic rays (GCRs) and solar energetic particles (SEPs) (Dartnell *et al.*, 2007a, 2010; Pavlov *et al.*, 2012). GCRs, produced outside the Solar System, are high-energy particles in energetic ranges from 10 MeV/nuc to more than 10 GeV/nuc. The GCR flux is modulated by the heliosphere and anticorrelated with solar activity (Hassler *et al.*, 2014), with a composition dominated by protons (85–90%) and helium ions (10–13%), although other minor components are present, such as electrons (1%) and heavier nuclei (1%) (Simpson, 1983). In contrast, SEPs are produced by the Sun, and its flux and composition are highly dependent on the solar cycle and other factors, making complex interactions with the GCRs within the heliosphere (Gloeckler, 1979). In general, SEP events generate particles characterized by lower energy particles than those involved in GCRs, and thus lack penetrating capabilities in planetary atmospheres and regolith. However, spontaneous SEP events can increase the particle energies with substantial fluxes reaching the surface, such as reported on the martian surface by the Radiation Assessment Detector (RAD) instrument aboard the NASA Mars Science Laboratory (MSL) (Hassler *et al.*, 2014).

Ionizing radiation can also penetrate meters into regolith or ice, and can cause structural and chemical changes on organic and biological molecules (Dartnell *et al.*, 2007b). For example, direct impacts by radiation produce fragmentation but not dissociation on the native structure of proteins (Miller *et al.*, 1998), and polysaccharides are depolymerized into small fragments after irradiating with gamma rays (Edwards *et al.*, 1977), and their antigenic properties are severely affected in a radiation dose-dependent manner (Csako *et al.*, 1987). In the case of nucleic acids, ionizing radiation is known to cause cross-linking and strand breaks in DNA (Rydberg, 1996) and RNA molecules (Hutchinson *et al.*, 1963).

In the context of planetary exploration, Dartnell *et al.* (2012) showed that prominent Raman spectral features of several biomolecules were substantially diminished after 15 kGy of irradiation, and by 150 kGy the spectra of carotenoid were completely destroyed. Pavlov *et al.* (2012), based on radiolysis constants determined from the gamma irradiation experiments on solid amino acid powders carried by other authors (Kminek and Bada, 2006), estimated that 100 amu organic molecules could be detectable at 4–5 cm beneath the surface of Mars even after 1 Gyr of exposure to both SEPs and GCRs, whereas the abundance of heavier organic molecules (300 amu and larger) would decrease 1000-fold due to ionizing radiation in <300 Myr. Filali-Mouhim *et al.* (1997) found that 70 kGy of radiation, equivalent to ∼1 Myr exposure beneath a meter of dry dust on Mars (Dartnell *et al.*, 2007b), would be sufficient to shatter lysozyme into multiple small fragments.

Despite these previous studies, and after decades of research on the effects of ionizing radiation on biological molecules involved in the immune response at the organismal level (Rao *et al.*, 2005; Joo *et al.*, 2015), on the killing of cancer cells (for a review see Bernier, 2016), on the loss of functionality of organic molecules (Hutchinson and Norcross, 1960; Augenstine, 1962; Butler and Robins, 1962; Orlova, 1993), on the radiolysis of small molecules (Kminek and Bada, 2006; Portugal *et al.*, 2014), and on the damage and fragmentation of biopolymers (Jabir *et al.*, 1989), it is still difficult to anticipate the effects of ionizing radiation on all types of biomolecules, because the extent of radiation damage depends on both the radiation dose and the type and size of the molecule. For example, Byun *et al.* (2000) reported a dramatic drop in the ability of the immunoglobulin E from allergy patients for immunoidentifying the shrimp heat-stable protein in a gamma radiation dose-dependent manner. They showed a direct correlation of protein fragmentation with the irradiation dose, from 0 to 10 kGy maximum. Radiation dose is, in turn, a function of exposure time and the radiation flux that reaches the molecule.

During the past two decades, immunosensors (bioaffinity-based biosensors using antibodies) have been proposed for *in situ* analysis for life detection in planetary exploration (Parro *et al.*, 2005, 2011b; Sims *et al.*, 2005; Sephton *et al.*, 2013). Studies on the stability of antibodies under the effect of ionizing radiation and other stresses demonstrated that their use in a potential mission to Mars is possible (Le Postollec *et al.*, 2009a; Baqué *et al.*, 2011, 2016; de Diego-Castilla *et al.*, 2011; Derveni *et al.*, 2012). However, it is also critical to understand how the ionizing radiation may affect the integrity of the potential molecular targets of the antibodies. In this study, taking the advantage of protein microarrays, we scale this approach up to a multiplex format to investigate the impact of long-term exposure to ionizing radiation on the immunoidentification of epitopes (the part of the target molecule where the antibody binds) on nonbiogenic and biogenic molecules.

Microarray and *in situ* synthetic technology (for a review see Liu *et al.*, 2012) allow testing the effect of radiation on tens of thousands of molecules simultaneously. Therefore, whole microorganisms (spores), macromolecules (*e.g.*, proteins, bacterial exopolymeric substances [EPS], and lipopolysaccharides [LPS]), and small molecules (amino acids, monosaccharides, carboxylic acid derivatives, and peptides) conjugated to proteins carriers (hapten conjugates) were exposed to ionizing radiation levels equivalent to 12,000 years, 0.6, and 6 Myr on the martian surface as inferred from the MSL data (Hassler *et al.*, 2014). These radiation doses and periods of time are relatively short in a planetary perspective; however, they are highly relevant for those scenarios where fresh material has been exposed to the unprotected surface such as the ejected material in the icy moons plumes (Parkinson *et al.*, 2007; McKay *et al.*, 2008) or recent material exposed in the martian surface (Schon and Head, 2012; Willmes *et al.*, 2012).

In previous work published by other authors and based on Monte Carlo simulations, it was reported that gamma and electrons account for the most abundant radiation fluxes on the surface of Mars with and without taking SEPs into account (Le Postollec *et al.*, 2009b). Therefore, the study of the effects of this type of radiation on the structural integrity of biochemicals can help to understand the long-term surviving ratio of organic molecules and potential biological polymers on the martian regolith. Our results indicate that although electron and gamma radiations severely affected the immunoidentification efficiency after several Myr exposure equivalent, a significant fraction of the irradiated target compounds could be still recognized by their corresponding antibodies.

## 2. Experimental Procedures

### 2.1. Selection of molecular targets

Twenty-nine organic compounds, from which 23 are biogenic, and spores from 2 bacterial strains with low (*Bacillus subtilis*) and high (*Streptomyces diastaticus*) G + C DNA content were used in this study (Table 1). Several proteins, peptides, amino acids (Cys and Tyr), LPSs, bacterial EPSs mainly composed of polysaccharides, a monosaccharide (*N*-acetyl-galactosamine), and small molecules involved in metabolic processes (coenzyme A and cAMP) were selected such as biochemical evidence of microbial life. All of these targets are universal in terrestrial biochemistry, including extremophilic microorganisms found in Mars analog environments (Parro *et al.*, 2011a).

**Table 1.** List and Description of the Compounds, Molecules and Antibodies Used in This Work

*Molecule/compound*	*Epitope (Da)*	*Ab name*	*Relevance*	*Reference*
Spores (*Bacillus subtilis*)^a^	Multiple. Unknown	IVH1C1	Resistant cell structure	Parro *et al.* (2011a)
Spores (*Streptomyces lividans*)	Multiple. Unknown	IVH2C1	Resistant cell structure	This work
EPS (*Dechloromonas agitata*)^a^	Multiple. Unknown	IVL6S2	Bacterial extracellular polymeric substances	This work
LPS-BSA (*Pseudomonas* sp.)^a^	Multiple. Unknown	Anti-LPS-BSA	Gram-negative bacterial cell wall	Parro *et al.* (2011a)
ABC transporter protein (*Thermus scotodoctus*)	Multiple. (70,000)	Anti-ABCtrans	ATP-binding translocase subunit	Rivas *et al.* (2011)
Streptavidin protein (*Streptomyces avidinii*)	Multiple. (60,000)	Anti-Stv	Bacterial protein	S6390 Sigma-Aldrich
GroEL protein (*Escherichia coli*)^a^	Multiple. (60,000)	Anti-GroEL	Heat shock chaperone	G6532 from Sigma-Aldrich
DsrB protein (*Archaeoglobus fulgidus*)^a^	Multiple. (42,000)	Anti-DsrB	Dissimilatory sulfite reductase B	Parro *et al.* (2011a)
FtsZ peptide-BSA (*Pseudomonas putida*)^a^	CPFEGRKRMQIADEGIR. (2006)	Anti-FtsZ	From FtsZ protein universal in the bacterial division	Parro *et al.* (2011a)
Rubredoxin peptide-BSA (*Desulfovibrio desulfuricans*)^a^	CHTQDETMKALEIKKDV. (1989)	Anti-RRO	From iron–sulfur protein involved in electron transfer in sulfur-metabolizing bacteria and archaea	Parro *et al.* (2011a)
DhnA2 peptide-BSA (*Nostoc* PCC73102)	SGRKAFQRPFEEGVKLC. (1952)	Anti-DhnA2	From fructose-biphosphate aldolase, involved in glycolysis, gluconeogenesis and the Calvin cycle	Parro *et al.* (2011a)
Bacterioferritin peptide-BSA (*D. desulfuricans*)^a^	CAENFAERIKELGGEP. (1762)	Anti-BFR	Iron storage protein	Parro *et al.* (2011a)
NifS2 peptide-KLH conjugate (*Leptospirillum ferrooxidans*)	LPVNKEGRVEIETLK. (1725)	A1496	From nitrogenase cofactor synthesis protein nifS	Fernández-Calvo *et al.* (2006)
ModA2 peptide-KLH conjugate (*L. ferrooxidans*)	MLAPLHKKIVYANTL. (1712)	A1494	From molybdenum transporter ModA	Fernández-Calvo *et al.* (2006)
GlnB1 peptide_KLH conjugate (*L. ferrooxidans*)	VEAIIKPFKLDAVKE. (1705)	A1492	Regulatory protein P-II (nitrogen metabolism)	Fernández-Calvo *et al.* (2006)
HscA1peptide-KLH conjugate (*L. ferrooxidans*)	SSLIPRNTTIPTQAK. (1626)	A1487	Chaperone protein HscA	Fernández-Calvo *et al.* (2006)
HscA2 peptide-KLH conjugate (*L. ferrooxidans*)	TFTIDANGILDVRAL. (1619)	A1488	Chaperone protein HscA	Fernández-Calvo *et al.* (2006)
ModA1 peptide-KLH conjugate (*L. ferrooxidans*)	PENSPQKTHVNVGIS. (1606)	A1494	From molybdenum transporter ModA	Fernández-Calvo *et al.* (2006)
NifH1peptide-KLH conjugate (*L. ferrooxidans*)	LAAEAGTVEDLEIED. (1574)	A1483	From Mo dinitrogenase reductase of the nitrogenase complex	Fernández-Calvo *et al.* (2006)
Coenzyme A-SH-BSA conjugate	CoA-SH. (767)	Anti-CoA	Universal in central metabolism	Parro *et al.* (2011a)
AEKAC peptide-BSA conjugate^a^	AEKAC. (520)	Anti-AEKAC	Bacterial cell wall structural peptide	Parro *et al.* (2011a)
Mellitic acid-BSA^a^	C_12_H_6_O_12_. (342)	Anti-mellitic	From oxidation of organic compounds	Blanco *et al.* (2013)
Cyclic AMP-BSA	cAMP. (329)	Anti-cAMP_N	Intracellular signal transductor	Parro *et al.* (2011a)
7-benzo-a-pyrene-C4-BSA^a^	C_20_H_12_. (252)	5G1^b^	Polyaromatic hydrocarbon	Karsunke *et al.* (2011)
6-benzo-a-pyrene-C4-BSA	C_20_H_12_. (252)	5G1^b^	Polyaromatic hydrocarbon	Karsunke *et al.* (2011)
*N*-acetyl-galactosamine-BSA^a^	*N*-acetyl-gal. (221)	Anti-NAG	Amino sugar	This work
Atrazine-OVA^a^	Atrazine. (215)	Anti-atrazine	Aromatic xenobiotic compound	This work
Tyrosine-BSA	Tyrosine. (181)	Anti-Tyr	Amino acid	Parro *et al.* (2011a)
*p-azo*-l-Phe-BSA^a^	l-Phe. (165)	Anti-Pazol^b^	Aromatic amino acid	Hofstetter *et al.* (2005)
4-(2-aminoethyl)-benzoic acid-BSA^a^	C_6_H_5_-COOH. (165)	Antibenzoic	From oxidative degradation of toluene	This work
Cys-BSA^a^	Cys. (121)	Anti-Cys	Amino acid	Parro *et al.* (2011a)

Compounds on the list are ordered according to epitope complexity and size (epitope-Da).

^a^ Molecules subjected to electron+gamma radiations.

^b^ Monoclonal antibody.

BSA = bovine serum albumin; EPS = exopolymeric substances; KLH = keyhole limpet hemocyanin; LPS = lipopolysaccharide; OVA = ovoalbumin.

Proteins and peptides were selected based on their involvement in universal metabolisms and cellular functions and their phylogenetic sequence preservation. Among them, we investigated 4 proteins and 12 peptides from proteins involved in transport across cell membrane (ABC transporter for potassium, ModA1 and ModA2 from a molybdenum transporter required for the nitrogenase and nitrogen fixation), energetic metabolisms (DhnA2 peptide), sulfur metabolism (DsrB and RRO), nitrogen metabolism (GlnB1, NifS2, and NifH1, from nitrogen regulator and nitrogenase components), universal iron storage system in bacteria (bacterioferritin), universal stress response proteins (GroEL, HscA1, and HscA2), cell septation and division (FtsZ), structural components (AEKAC peptide), and streptavidin (a microbial protein extensively used as a tool in molecular biology. It binds the vitamin biotin, an enzyme cofactor indispensable to metabolic fixation of carbon dioxide). All these molecules are involved in metabolisms widely spread among the known microbial life on Earth.

They are potential targets for life detection systems in planetary exploration under development (Parro *et al.*, 2011b). We also investigated aromatic compounds such as a modified amino acid (*p-azo*-l-Phe) and four small molecules [mellitic acid, 7 and 6-benzo-a-pyrene-C4, and 4-(2-aminoethyl)-benzoic acid], as stable intermediates of what might be expected on Mars after the oxidation of organic compounds either biogenic or nonbiogenic (Benner *et al.*, 2000). Although atrazine (an herbicide) is not a biomarker, it was selected as an example of small aromatic architecture and xenobiotic compound (synthetically produced and usually not found in nature. It is not expected to be found on Mars and we normally use it as an internal positive control of the assay).

All the peptides and small molecules (generally called “haptens” in the immunological terminology) were bound to large carrier proteins such as bovine serum albumin (BSA), keyhole limpet hemocyanin (KLH), or ovoalbumin (OVA), to obtain the corresponding hapten conjugate. Immunogenic hapten conjugates were used for eliciting an immune response and ensuring a high-yield antibody production against the hapten. For immunoassay, the hapten conjugates were immobilized to epoxy-activated glass slides (through primary amines) to expose the haptens (the molecular targets) to the antibodies.

Small molecules, in general, are not at all or badly recognized by the antibodies when they are directly immobilized onto a solid surface, simply because the antibodies cannot access the whole recognizable structure (epitope) on the molecule due to steric hindrance. The immobilized hapten conjugate ensures the direct exposure of the hapten to the radiation while allowing the antibody access to the intact or radiation damaged hapten. This is not an issue for detecting small molecules in natural samples, because hapten and other analytes are in solution and they are easily accessible by the antibody through a competitive or inhibitory immunoassay (Fernández-Calvo *et al.*, 2006).

### 2.2. Sample preparation for irradiation experiments

**FIG. 1.**
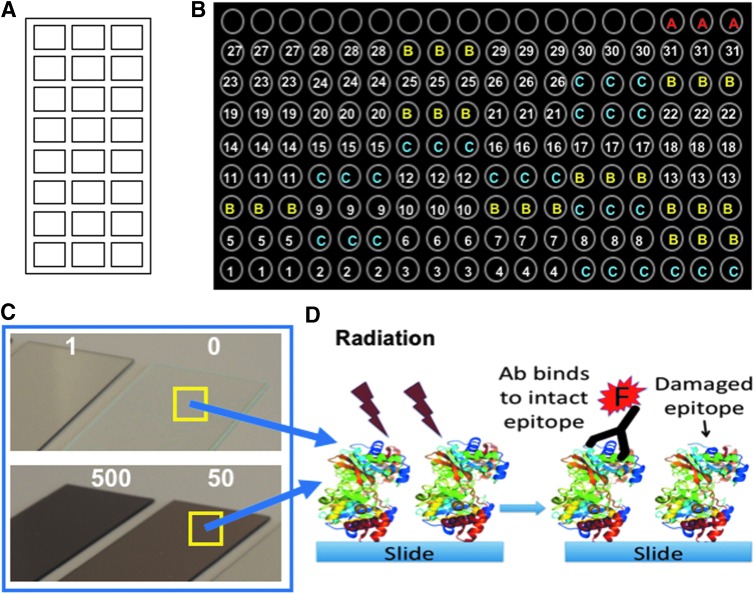
Experimental setup for irradiation of multiple compounds simultaneously. **(A)** Several biological and organic compounds (*e.g.*, spores, proteins, peptides, and EPS) were printed (150 μm diameter spots) onto microscope slides in 24 microarray replicates per slide. This format allowed to assay up to 11 different antibodies by duplicate. **(B)** Schematic of a printing pattern layout (by triplicate) of the biomolecules: *A*, fluorescent preimmune antibody as a frame reference; *B*, printing buffer only; *C*, BSA; 1–31 (by triplicate spots), each of the biomolecules in Table 1 [1: GroEL, 2: ABC transporter, 3: DsrB, 4: *Bacillus subtilis* spores, 5: NifH1-KLH, 6: HscA1-KLH, 7: ModA2-KLH, 8: NifS2-KLH, 9: rubredoxin-BSA, 10: FtsZ-BSA, 11: lipopolysaccharide-BSA, 12: Cys-BSA, 13: HscA2-BSA, 14: *p-azo*-l-Phe-BSA, 15: DhnA2-BSA, 16: atrazine-OVA, 17: Tyr-BSA, 18: BFR-BSA, 19: *Streptomyces diastaticus* spores, 20: ModA1-KLH, 21: GlnB1-KLH, 22: 7-benzo-a-pyrene-C4-BSA, 23: EPS from *Dechloromonas agitata*, 24: 4-(2-aminoethyl)benzoic acid-BSA, 25: AEKAC-BSA, 26: 6-benzo-a-pyrene-C4-BSA, 27: cAMP-BSA, 28: streptavidin, 29: CoA-BSA, 30: mellitic acid-BSA, 31: *N*-acetyl-galactosamine-BSA]. **(C)** Darkening effect of the microscope slides: before radiation (0) and after exposure to 1 kGy (1), 50 kGy (50), and 500 kGy (500) radiation doses. **(D)** Schematic explaining a hypothetical radiation effect on the epitopes at molecular level and how it can alter the binding of fluorescent (F) antibodies. BSA, bovine serum albumin; EPSs, exopolymeric substances; KLH, keyhole limpet hemocyanin; OVA, ovoalbumin.

Diagnostic organic molecules and two types of spore-forming bacteria were immobilized in 150 μm diameter spots on epoxy-activated glass slides as previously described (Blanco *et al.*, 2013) (Fig. 1). In brief, (i) compounds at 0.8 mg/mL were prepared in 1 × commercial protein printing buffer (Whatman; Schleicher & Schuell, Sandford, ME) and 0.01% Tween 20 as spotting solution and (ii) printing was done in a triplicate spot pattern for 24 arrays per slide using a MicroGridII arrayer (Genomic Solutions). This format allowed us to assay up to 11 different antibodies per duplicate. A schematic of the printing pattern layout is shown in Figure 1B. Three sets of slides were prepared for (i) electron radiation exposure, (ii) gamma radiation exposure, and (iii) electron+gamma radiation exposure. Slides exposed to electron+gamma radiations contained only a subset of all the molecules tested.

The slides were left to dry at room temperature and then transported to radiation facilities. Every spot was the result of spotting about 7–10 nL of compound in printing buffer. After dried, a <10 μm thick layer with the compound and buffer salts was created over the truly immobilized molecule “monolayer” on the slide. Assuming a spot thickness <10 μm, differences in penetration capabilities of both types of radiation were considered negligible. Attenuation value has been calculated by simple physics and the result is <1%.

### 2.3. Radiation treatments

Three sets of slides containing the immobilized compounds (Table 1) were directly exposed to 0 kGy (control), 1, 50, and 500 kGy radiation doses (Table 2). One set of slides was exposed to these doses of electron radiation only with 10 MeV of energy at Ionisos Ibérica (Tarancón, Spain) with a Rhodotron tt200an electron accelerator, at room temperature (c.a. 20.5°C) in air and at atmospheric pressure. Irradiation produces an increase of the sample temperature, ∼0.5°C every kGy applied. To minimize the effect of temperature on the printed compounds at high doses, irradiation was applied at 50 kGy/s with 10-min intervals to allow heat dissipation to room temperature. In this way, the maximal temperature in the 500 kGy samples was 50°C for about 10 s at most. A second set of slides was exposed to the same doses of gamma radiation only with a ^60^Co radionuclide 3 MeV gamma ray source at the Unidad Náyade of CIEMAT (CSIC, Madrid, Spain) with a dose rate of 5.46 kGy/h again under atmospheric conditions, room temperature 19°C.

**Table 2.** Irradiation Conditions Used in This Study

*Radiation type*	*Radiation dose (kGy)*	*Equipment*	*Energy (MeV)*	*Dose rate (kGy/h)*	*Irradiation time (h)*	*Sample*^a^*temperature (°C)*
Electrons	1	Rhodotron tt200an accelerator. Ionisos Iberica	10	3600	0.00027	20.5
	50			180,000	0.00027	45
	500			180,000	0.0027	50
Gamma	1	^60^Co. CIEMAT (CSIC)	3	5.46	0.183	19
	50			5.46	9.157	19
	500			5.46	91.571	19
Electrons + gamma	0.5 + 0.5	Van de Graaff accelerator+^60^Co. CIEMAT (CSIC)	2, 3	111.6, 4.97	0.004 + 0.1	24, 19
	25 + 25			111.6, 4.97	0.223 + 5.03	24, 19
	250 + 250			111.6, 4.97	2.23 + 50.3	24, 19

^a^ Temperature reached by the microscope slide after total dose irradiation and time.

A third set of slides with 16 compounds given in Table 1 [spores from *B. subtilis*, EPS, LPS-BSA, ABC transporter, GroEL, DsrB, BFR-BSA, rubredoxin-BSA, FtsZ-BSA, AEKAC-BSA, cAMP-BSA, 7-benzo-a-pyrene-C4-BSA, *N*-acetyl-galactosamine-BSA, atrazine-OVA, *p-azo*-l-phealanine-BSA, 4-(2-aminoethyl)benzoic acid-BSA, and cysteine-BSA] was exposed to both radiations consecutively at the CIEMAT (CSIC) facilities. To keep the same doses as mentioned, slides were first irradiated with 0.5, 25, and 250 kGy with an electron accelerator (Van de Graaff accelerator) operating with 2 MeV at 23°C ambient temperature, and then irradiated with 0.5, 25, and 250 kGy of gamma radiation with a ^60^Co source at a dose rate of 4.97 kGy/h and 3 MeV at 19°C room temperature. The absorbed doses were computed based on absorbance measurements of radiocromic and Perpex dosimeters by spectrophotometry, where the dosimeters were previously characterized for the trial conditions. The irradiation procedure was performed at atmospheric pressure (1 atm), ambient temperature (always <25°C), and under air. Constraints in the equipment and facilities did not allow carrying out this procedure to emulate other atmospheric conditions, such as the martian environment.

The production of oxygenated radicals formed by the action of ionizing radiation on water (Hutchinson *et al.*, 1963) and on air containing oxygen (ozonolysis) is well known. To avoid side effects due to the damage produced by the free radicals formed from the water, all the experiments were done with dry samples. All the slides were preserved under a silica desiccant until irradiation. In addition, it is expected that most of the potential biomolecules in the martian regolith are likely to be desiccated or frozen, always with very little humidity. Consequently, because the irradiation was performed under air, we considered these conditions as a worst-case scenario where the radiation effect might have been increased by secondary events such as ozonolysis.

### 2.4. Antibody production, purification, and labeling

The antibodies used in the work are part of the antibody collection of the Centro de Astrobiologia or from some collaborators (Table 1). Most of the antibodies were polyclonal, produced in rabbit, affinity purified with protein A, and fluorescently labeled with Alexa-Fluor 647 as previously reported (Rivas *et al.*, 2008; Blanco *et al.*, 2015).

### 2.5. Fluorescent immunoassays

The interaction between any antigen, analyte, and epitope with its antibody is mediated by noncovalent forces as electrostatic, ionic, van der Waals, hydrogen bonds, or hydrophobic interactions (Janeway *et al.*, 2001). Any structural, chemical, or conformational change in the antigen or in the antibody binding site (epitope) that modify the mentioned interaction forces may mildly or severely impair the strength of the antibody binding or even avoid any specific interaction (Davies and Cohen, 1996). We took the advantage of these antibody properties by assuming that any damage produced by radiation on the structure, chemistry, or electrostatic state of the target molecules (particularly on the epitopes) may impair or completely abolish the binding of the fluorescent antibodies. Therefore, after irradiation, direct immunoassays were carried out as an evaluation test of the integrity or the degree of alteration of the epitopes (again, the parts of the molecules recognized by the antibody) in the printed biomolecules. After immunoassays, the fluorescence intensity at each spot was quantified and plotted. Because reporter fluorescent antibodies can only bind to their corresponding antigenic molecules if the later still keep intact or slight structurally altered epitopes, the higher the fluorescence intensity of each spot on the array the lower the amount of damage of the epitopes after irradiation. The absence of fluorescence is indicative that the epitopes have suffered any structural, chemical, or conformational alteration that precludes the antibody binding.

Immunoassays were performed as follows: after blocking all free epoxy groups on the slides by saturating their binding capacity with BSA, and removing the excess of noncovalently bound molecules with 0.5 M Tris-HCl pH 9, 5% BSA for 5 min, and 0.5 M Tris-HCl pH 8, 2% BSA for 30 min, the slides were dried by a short centrifugation. Then, 50 μL of each corresponding Alexa 647-labeled antibody (all of them at a concentration of 2 μg/mL, except anti-*p-azo*-l-phenylalanine antibody whose working concentration was 0.1 μg/mL) was incubated with the microarray for 1 h at room temperature. Then, slides were washed with Tris Buffered Saline with Tween and double Reinforced RR (TBSTRR) buffer (0.4 M Tris-HCl pH 8, 0.3 M NaCl, 0.1% Tween 20), dried as mentioned, and scanned for fluorescence at 635 nm in a GenePix 4100A scanner (Genomic Solutions). Images were analyzed and the fluorescence was quantified by using the GenePix Pro Software as described (Parro *et al.*, 2011a; Blanco *et al.*, 2015). All pairs molecule (epitope)-antibody were assayed by duplicate and the fluorescence intensity was the average of six replicated spots (three per array and per molecule tested).

### 2.6. Statistical analysis of the fluorescence intensity values

The data of the fluorescence intensity units were analyzed statistically with two-way ANOVA, *post hoc* multiple comparison Tamhane's T2 tests, and two-sample tests of proportions at a significance level of 0.05. Tamhane's T2 tests were used to search for statistical differences among the three radiation doses for each type of radiation, whereas two-sample test of proportions was used to study the statistical differences between the three types of radiation as a function of the radiation dose.

### 2.7. Computation of radiolysis rates

We estimated the radiolysis rates of the molecules after irradiation by two different radiolysis models. The first one is a simple exponential model, previously described by Kminek and Bada (2006), which considers a single decomposition process:

\begin{align*}
 \frac { N }  { { { N_0 } } } = \; { e^ { - { k_r } D } } , \tag { { \rm Eq } . \ 1 } 
\end{align*}

where *N/N*_0_ was the surviving ratio, which in this particular case was obtained by the measurement of the fluorescence intensity after the immunoassay of the irradiated molecule with its corresponding fluorescent antibody, *k_r_* was the radiolysis rate in kGy^−1^, and *D* the radiation dose in kGy. The second model considers a decomposition through two chemical pathways:

\begin{align*}
 \frac { N }  { { { N_0 } } } = \;A* { e^ { - { k_1 } D } } + \;B* { e^ { - { k_2 } D } } , \tag { { \rm Eq } . \ 2 } 
\end{align*}

where each pathway has its own radiolysis rate, *k*_1_ and *k*_2_, in kGy^−1^, and the coefficients *A* and *B* are the contributions to the total decomposition. The model coefficients were computed by means of linear regression in the case of the simple exponential model (Eq. 1), and by nonlinear regression in the case of the two pathways model (Eq. 2). A larger number of irradiation doses would be desirable for a more accurate model fit, particularly for the two pathways model. Both models have been computed using the average decomposition data obtained for each kind of irradiation. In addition, a model taking into account the average values obtained for all kinds of radiations has also been computed. Only molecules with all the *N/N*_0_ ratio measurements higher than 2% were considered for computation for each radiation type, and, therefore, model results are not strictly comparable.

### 2.8. Applicability to Mars

The radiation doses utilized in our experiments were selected to represent radiation exposure times equivalent to 12,000 years, 0.6, and 6 Myr on the martian surface, based on the simulated results presented by Hassler *et al.* (2014) using the surface measurements by NASA's MSL mission. This particular choice reflects the expected threshold of survival of complex organic molecules in the martian environment based on previous experiments (Dartnell *et al.*, 2012), or the radiation exposition times for relatively freshly exposed material in planetary surfaces as Enceladus plumes, recent martian impacts (Schon and Head, 2012), or recent scenarios for potential biological activity under the martian permafrost (McKay *et al.*, 2013).

Our results allow for estimation of the target survival ratio (*N/N*_0_) as a function of the martian regolith depth, for illustrative exposure times at the MSL landing site. The *N/N*_0_ ratio was computed by the previously considered models, described by means of Equations 1 and 2, using the model parameters for an average radiation field. The simulations included the disproportional damage by higher LET (linear energy transfer) radiation present on Mars and not experimented in this work, such as protons or HZE (high atomic number and energy) ions. Further research, already on progress, is focused on evaluating the damage by other types of radiation such as protons at different energies and doses.

## 3. Results

### 3.1. Exposure of biochemical and organic compounds to ionizing radiation

**FIG. 2.**
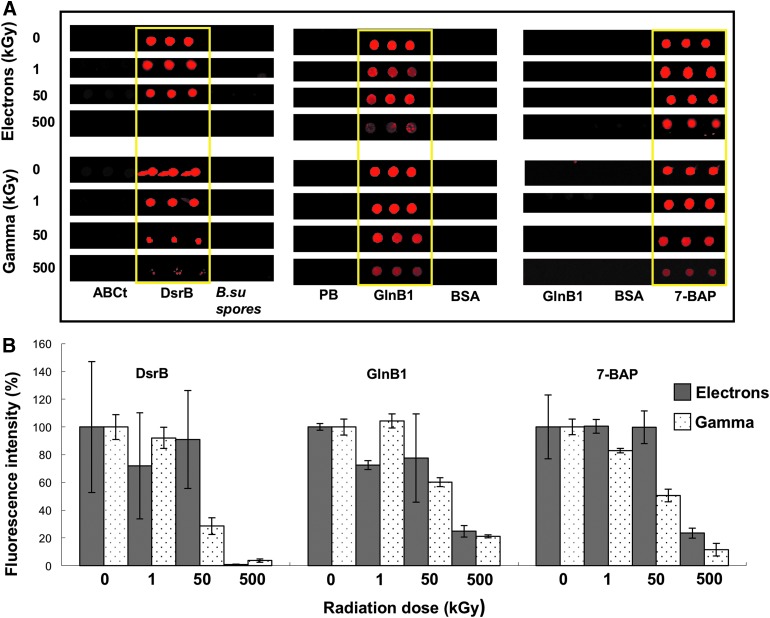
Gamma and electron radiations impair the immunoidentification of the target compounds. Examples of the immunoidentification response of three types of molecules and their antibodies: a protein (DsrB), a peptide (GlnB1), and a PAH (7-benzo-a-pyrene-C4-BSA), after being irradiated with 1, 50, and 500 kGy. **(A)** Fluorescent images of the spots on the microarray after direct fluorescent immunoassay with their corresponding antibodies (Table 1). Negative control spots (black) containing PB, BSA, and other molecules not expected to react in these assays are shown: those corresponding to ABC transporter, *B. subtilis* spores, or GlnB1 when immunoassay was done with anti-DsrB or anti-7-BAP antibodies, respectively (left and right pannels in A). **(B)** The fluorescence intensity resulted from the immunoassays after different radiation doses (1, 50, and 500 kGy) was quantified and plotted as a function of the nonirradiated control, which corresponded to 100% (0, radiation dose). Filled gray bars correspond to electron radiation and dotted bars to gamma radiation. PB, printing buffer; BSA, bovine serum albumin.

Strong ionizing radiation on thin atmosphere planetary bodies can modify and destroy potential molecular biomarkers and affect their detection with *in situ* analytical methods. To investigate the effects of ionizing radiation on the stability of biological polymers and organic molecules, a set of biological (including bacterial spores) and organic compounds (conjugated to proteins) were immobilized onto glass slides and subjected to several doses of electron and gamma radiations (Fig. 1; Tables 1 and 2) at atmospheric pressure and room temperature (20°C). Exposure of target compounds to ionizing radiation caused a loss of the immunoidentification signal (*i.e.*, loss of fluorescence after direct immunoassay), mostly due to epitope damage (Fig. 2).

**FIG. 3.**
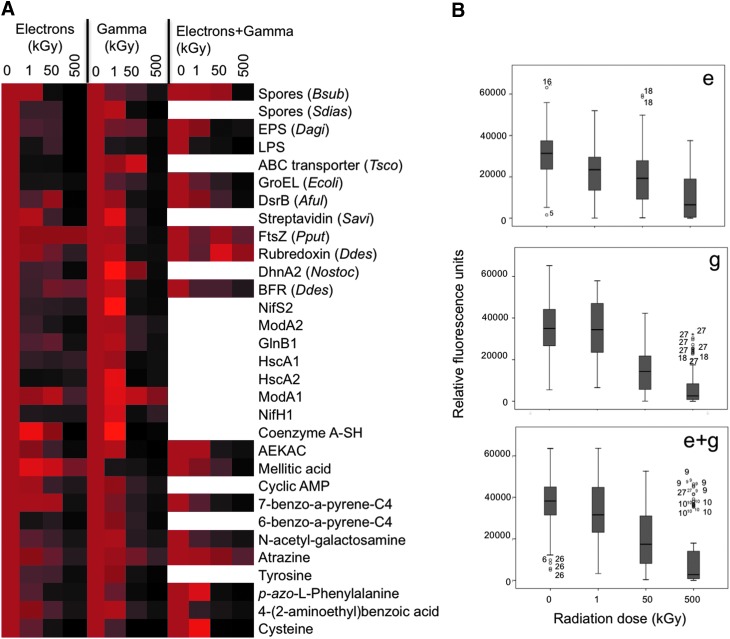
Epitope damage by electron and gamma radiations. **(A)** Data from Tables 3–5, corresponding to electron only, gamma radiation only, and electrons+gamma, were plotted in a heatmap for a graphical pattern visualization. From bright red (highest fluorescence signal) to black (0%), the color scale shows the percentage of the antibody binding after different radiation doses (1, 50, and 500 kGy), considering 100% the values from nonirradiated control. For some molecules, the highest intensity was obtained after an exposure to 1 or 50 kGy (see text for explanation). **(B)** Statistical ANOVA with all fluorescence data from electron (e), gamma (g), and electrons+gamma (e+g) irradiated sets. The boxes show the median, Q1, Q3, and intervals of the fluorescence values of all of the molecules on the microarray per radiation dose. Each group of values came from 6 or 12 spots per compound/molecule, 3 per array, that is 23 compounds × 6 spots means 138 data values plus 8 compounds × 12 spots means 96, then, 234 values for each electron radiation dose and 30 × 6, corresponding to 180 data values for each gamma radiation dose, and in the case of e+g where the figures were 16 × 6, corresponding to 96 data values for each dose. Compounds are ordered according to epitope complexity and size.

A heatmap representation of the relative fluorescence values for all compounds (Fig. 3A) showed a clear negative effect of the different irradiation experiments on the immunoidentification by the corresponding antibody. Results of the two-way ANOVA reveal statistically significant differences in the loss of immunoidentification as a function of radiation dose, and this effect was different for each type of radiation assayed (Fig. 3B). Tamhane's T2 tests were performed to test the effect of the dose for each type of radiation. The average fluorescence intensity for each molecule at every dose was relativized with respect to the nonirradiated control, considered as 100% of the immunoidentification signal (Tables 3–5).

**Table 3.** Relative Fluorescence Signal—Normalized to the Nonirradiated Control as 100%—of the Tested Compounds After Electron Irradiation

	*Radiation dose (kGy)*			
*Compound/molecule*	*0*	*1*	*50*	*500*
*B. subtilis* spores	100 ± 50.5 (a)	100.1 ± 18 (ab)	16.3 ± 9 (a)	0 ± 0 (b)
*Streptomyces diastaticus* spores	100 ± 3.7 (a)	62.5 ± 4.4 (b)	63.2 ± 17 (b)	0 ± 0 (c)
EPS (*D. agitata*)	100 ± 25.7 (a)	71.4 ± 24.2 (a)	66.3 ± 24.6 (a)	0 ± 0 (b)
LPS	100 ± 40.5 (a)	31.1 ± 2.9 (a)	64.6 ± 27 (a)	0 ± 0 (b)
ABC transporter (*T. scotodoctus*)	100 ± 70.7 (a)	20.3 ± 17 (b)	16.8 ± 13.9 (b)	0.5 ± 0.1 (c)
GroEL (*E. coli*)	100 ± 18.2 (a)	30.5 ± 25.8 (b)	33.7 ± 31.9 (b)	9.5 ± 1.6 (b)
DsrB (*A. fulgidus*)	100 ± 47.2 (a)	72 ± 38.2 (a)	91 ± 35.3 (a)	0.8 ± 0.2 (b)
Streptavidin (*S. avidinii*)	100 ± 6.3 (a)	104.5 ± 9.5 (a)	64.8 ± 13.1 (b)	2.2 ± 0.4 (c)
FtsZ (*P. putida*)	100 ± 3.7 (a)	88 ± 5.7 (b)	87.9 ± 5.5 (b)	87.9 ± 5.4 (b)
Rubredoxin-BSA (*D. desulfuricans*)	100 ± 14 (a)	91.5 ± 7.8 (a)	80 ± 6.5 (b)	54.8 ± 16.3 (c)
DhnA2 (*Nostoc* PCC73102)	100 ± 13.6 (a)	66 ± 10.6 (b)	69.4 ± 3.9 (b)	5.7 ± 3.2 (c)
BFR (*D. desulfuricans*)	100 ± 3.9 (a)	63.7 ± 18.4 (b)	81.1 ± 1.2 (b)	79 ± 1.8 (b)
NifS2 (*L. ferrooxidans*)	100 ± 8.9 (a)	57.2 ± 8.84 (b)	53.2 ± 7.8 (bc)	47 ± 6.4 (c)
ModA2 (*L. ferrooxidans*)	100 ± 23.1 (a)	63.3 ± 68.1 (ab)	40.4 ± 20.5 (b)	7.9 ± 5.3 (b)
GlnB1 (*L. ferrooxidans*)	100 ± 2.4 (a)	72.5 ± 3.3 (b)	77.7 ± 31.8 (abc)	24.9 ± 4.1 (c)
HscA1 (*L. ferrooxidans*)	100 ± 13.3 (a)	57.9 ± 10.5 (b)	51.2 ± 10 (b)	58.3 ± 11 (b)
HscA2 (*L. ferrooxidans*)	100 ± 50.6 (a)	16.6 ± 10 (b)	20.6 ± 11 (b)	39.3 ± 14.7 (c)
ModA1 (*L. ferrooxidans*)	100 ± 5.6 (a)	90.6 ± 14.4 (ab)	105.4 ± 16.5 (a)	68 ± 8 (b)
NifH1 (*L. ferrooxidans*)	100 ± 17.7 (a)	46.3 ± 2.6 (b)	39.5 ± 8 (b)	38.4 ± 5.7 (b)
Coenzyme A-SH	100 ± 51.7 (a)	129.2 ± 12.5 (a)	86.7 ± 52.1 (ab)	0 ± 0 (b)
AEKAC	100 ± 0.6 (a)	85.5 ± 1.4 (b)	64.4 ± 3.7 (c)	7.2 ± 1.6 (d)
Mellitic acid	100 ± 4.8 (a)	123.7 ± 14.2 (b)	114.4 ± 20.2 (abc)	82.5 ± 2.7 (c)
Cyclic AMP	100 ± 1.8 (a)	97.2 ± 1.4 (a)	62.3 ± 3 (b)	44.5 ± 0.5 (c)
7-Benzo-a-pyrene-C4	100 ± 22.9 (a)	100.5 ± 5 (a)	99.8 ± 11.9 (a)	23.4 ± 3.6 (b)
6-Benzo-a-pyrene-C4	100 ± 25.3 (a)	34.2 ± 4.3 (b)	50.3 ± 12.7 (b)	4.1 ± 0.7 (c)
*N*-acetyl-galactosamine	100 ± 10.5 (a)	75.7 ± 2.1 (b)	64.4 ± 9.9 (b)	34.8 ± 1.3 (c)
Atrazine	100 ± 5.8 (a)	86 ± 4 (b)	79.1 ± 1.5 (c)	49.1 ± 2.4 (d)
Tyrosine	100 ± 3.6 (a)	69.5 ± 2.9 (b)	67.1 ± 6.2 (b)	13.8 ± 2.5 (c)
*p-Azo*-l-Phe	100 ± 12 (a)	73.6 ± 28.8 (ab)	26.5 ± 9.1 (bc)	23 ± 4.2 (c)
4-(2-aminoethyl)benzoic acid	100 ± 2.7 (a)	86.1 ± 2.5 (b)	71 ± 1.5 (c)	34.2 ± 1.7 (d)
Cysteine	100 ± 5.8 (a)	77.3 ± 24.9 (a)	26.8 ± 1.9 (b)	0.4 ± 0.1 (c)

Errors were estimated as the standard deviation of six independent fluorescence measurements, except for GroEL, DsrB, streptavidin, FtsZ, rubredoxin, NifS2, HscA1, and HscA2 where estimation was done with 12 fluorescence measurements.

Letters in brackets represent statistical significant differences with respect to the control (0 kGy), that is, samples sharing the same letter are not significantly different (*p* ≥ 0.05) based on Tamhane's T2 multiple range tests. Compounds in the table are ordered according to epitope complexity and size.

**Table 4.** Relative Fluorescence Signal—Normalized to the Nonirradiated Control as 100%—of the Tested Compounds After Gamma Irradiation

	*Radiation dose (kGy)*			
*Compound/molecule*	*0*	*1*	*50*	*500*
*B. subtilis* spores	100 ± 36.3 (a)	77.5 ± 34.2 (ab)	67.6 ± 20.2 (a)	28.3 ± 6.5 (b)
*S. diastaticus* spores	100 ± 12.4 (a)	106.9 ± 13 (a)	12.7 ± 2.4 (b)	0 ± 0 (c)
EPS (*D. agitata*)	100 ± 11.1 (a)	80.5 ± 6.9 (b)	79.2 ± 9.7 (b)	9.2 ± 1.6 (c)
LPS	100 ± 22.8 (a)	43.5 ± 15.5 (b)	33 ± 9.6 (b)	2.8 ± 0.7 (c)
ABC transporter (*T. scotodoctus*)	100 ± 11.4 (ab)	91.9 ± 5.3 (a)	115.4 ± 13.9 (b)	3.1 ± 0.4 (c)
GroEL (*E. coli*)	100 ± 9.3 (a)	74.2 ± 4.2 (b)	60.1 ± 3.6 (c)	9.5 ± 0.4 (d)
DsrB (*A. fulgidus*)	100 ± 8.9 (a)	92.1 ± 7.6 (a)	28.6 ± 6 (b)	3.8 ± 1.2 (c)
Streptavidin (*S. avidinii*)	100 ± 5.2 (a)	125 ± 12.1 (b)	53.3 ± 13.9 (c)	2.3 ± 0.3 (d)
FtsZ (*P. putida*)	100 ± 1.8 (a)	98.3 ± 3.4 (a)	46.6 ± 4.2 (b)	8.9 ± 1.1 (c)
Rubredoxin-BSA (*D. desulfuricans*)	100 ± 2.7 (a)	104.9 ± 2.8 (a)	23.9 ± 8.9 (b)	18.5 ± 2.3 (b)
DhnA2 (*Nostoc* PCC73102)	100 ± 54.9 (ab)	138.8 ± 3.3 (b)	85.4 ± 1.7 (a)	9.6 ± 3 (c)
BFR (*D. desulfuricans*)	100 ± 2.4 (a)	110.4 ± 2.5 (b)	29.9 ± 0.8 (c)	4.7 ± 0.5 (d)
NifS2 (*L. ferrooxidans*)	100 ± 9.3 (a)	140.5 ± 8.3 (b)	21.7 ± 1.9 (c)	7 ± 0.9 (d)
ModA2 (*L. ferrooxidans*)	100 ± 4.3 (a)	98.9 ± 7.4 (a)	59.5 ± 6.5 (b)	36.6 ± 2.2 (c)
GlnB1 (*L. ferrooxidans*)	100 ± 5.8 (a)	104.3 ± 5 (a)	60.2 ± 3.2 (b)	21.1 ± 1 (c)
HscA1 (*L. ferrooxidans*)	100 ± 8 (a)	94.4 ± 4.7 (a)	9.4 ± 3.8 (b)	23 ± 1.7 (c)
HscA2 (*L. ferrooxidans*)	100 ± 3.2 (a)	125.9 ± 3.4 (b)	2.2 ± 0.4 (c)	23.0 ± 4.3 (d)
ModA1 (*L. ferrooxidans*)	100 ± 12.6 (ac)	123.9 ± 2.1 (b)	109.5 ± 14.7 (ab)	84.9 ± 4.4 (c)
NifH1 (*L. ferrooxidans*)	100 ± 23.9 (a)	119.4 ± 16.3 (a)	17.1 ± 3.2 (b)	57.4 ± 14.5 (b)
Coenzyme A-SH	100 ± 11.7	154.3 ± 22.4	N.D.	2.5 ± 0.7
AEKAC	100 ± 1.3 (a)	104.3 ± 3 (a)	3.3 ± 0.3 (b)	0 ± 0 (c)
Mellitic acid	100 ± 10.1 (a)	37.8 ± 8.5 (b)	33.5 ± 1.2 (b)	3.3 ± 0.5 (c)
Cyclic AMP	100 ± 3.8 (a)	88.5 ± 1.4 (b)	58.7 ± 0.9 (c)	29.7 ± 0.7 (d)
7-Benzo-a-pyrene-C4	100 ± 5.7 (a)	82.9 ± 1.6 (b)	50.7 ± 4.5 (c)	11.6 ± 4.5 (d)
6-Benzo-a-pyrene-C4	100 ± 5.1 (a)	85.6 ± 2 (b)	54.5 ± 1.9 (c)	3.8 ± 0.3 (d)
*N*-acetyl-galactosamine	100 ± 3.4 (a)	101.2 ± 11.8 (a)	51 ± 2.5 (b)	32.2 ± 4.8 (c)
Atrazine	100 ± 7 (a)	93.5 ± 5 (ab)	81.1 ± 7.5 (b)	59.7 ± 9.1 (c)
Tyrosine	100 ± 14.2 (a)	87.3 ± 7.1 (a)	25.2 ± 3 (b)	1.47 ± 0.2 (c)
*p-Azo*-l-Phe	100 ± 8 (a)	78.8 ± 4.4 (b)	9.4 ± 1.4 (c)	0 ± 0 (d)
4-(2-Aminoethyl)benzoic acid	100 ± 5.2 (a)	102.9 ± 2.4 (a)	54.9 ± 4.2 (b)	21.2 ± 3.9 (c)
Cysteine	100 ± 9.2 (a)	68.1 ± 15.3 (b)	0.1 ± 0 (c)	0 ± 0 (d)

Errors were estimated as the standard deviation of six independent fluorescence measurements.

Letters in brackets represent statistical differences with respect to the control (0 kGy) revealed by Tamhane's T2 tests at a significance level of 0.05, that is, samples sharing the same letter are not significantly different (*p* ≥ 0.05) based on Tamhane's T2 multiple range tests. Compounds in the table are ordered according to epitope complexity and size.

**Table 5.** Relative Fluorescence Signal—Normalized to the Nonirradiated Control as 100%—of the Tested Compounds After Gamma+Electron Irradiations

	*Radiation dose (kGy)*			
*Compound/molecule*	*0*	*1*	*50*	*500*
*B. subtilis* spores	100 ± 7 (a)	98.9 ± 3 (a)	92.1 ± 8.8 (a)	5.1 ± 0.6 (b)
EPS (*D. agitata*)	100 ± 13.5 (a)	85.7 ± 12.1 (a)	12.8 ± 5 (b)	24.4 ± 2.7 (c)
LPS	100 ± 37 (a)	37.5 ± 15.3 (b)	18.9 ± 13 (bc)	0 ± 0 (c)
GroEL (*E. coli*)	100 ± 46.5 (a)	77.2 ± 59 (ab)	54.4 ± 10.7 (a)	3 ± 0.3 (b)
DsrB (*A. fulgidus*)	100 ± 2.6 (a)	85.6 ± 6.9 (b)	66.7 ± 2.6 (c)	5.8 ± 0.6 (d)
FtsZ (*P. putida*)	100 ± 8.7 (a)	78.4 ± 2.2 (b)	92.2 ± 2.7 (b)	77.5 ± 6.9 (c)
Rubredoxin-BSA (*D. desulfuricans*)	100 ± 2.3 (a)	76.3 ± 4.3 (b)	121.1 ± 1.7 (c)	90 ± 1.5 (d)
BFR (*D. desulfuricans*)	100 ± 1.7 (a)	69.1 ± 2.6 (b)	68.9 ± 1.1 (a)	47 ± 3.8 (b)
AEKAC	100 ± 1.3 (a)	100.3 ± 1.3 (a)	38.2 ± 5.3 (b)	19.3 ± 1.6 (c)
Mellitic acid	100 ± 5 (a)	84.6 ± 6.6 (b)	67.3 ± 6.6 (c)	3 ± 2.5 (d)
7-Benzo-a-pyrene-C4	100 ± 2.2 (a)	74.1 ± 0.5 (b)	34.6 ± 1.4 (c)	3.9 ± 0.2 (d)
*N*-acetyl-galactosamine	100 ± 2.1 (a)	68.9 ± 3.5 (b)	51.1 ± 9.8 (c)	28.5 ± 11.7 (d)
Atrazine	100 ± 1.3 (a)	97.1 ± 8.4 (ab)	85.4 ± 4.6 (b)	73.6 ± 4 (c)
*p-Azo*-l-Phe	100 ± 4.1 (a)	121.2 ± 7.8 (b)	15 ± 3.8 (c)	1.2 ± 0.5 (d)
4-(2-Aminoethyl)benzoic acid	100 ± 14.4 (a)	57.4 ± 8.5 (b)	37.3 ± 2.9 (c)	14.8 ± 9.7 (d)
Cysteine	100 ± 4.7 (a)	129.7 ± 8.4 (b)	0.8 ± 0.1 (c)	0.1 ± 0 (d)

Errors were estimated as the standard deviation of six independent fluorescence measurements.

Letters in brackets represent statistical differences with respect to the control (0 kGy), that is, samples sharing the same letter are not significantly different (*p* ≥ 0.05) based on Tamhane's T2 multiple range tests. Compounds in the table are ordered according to epitope complexity and size.

### 3.2. Electron radiation exposure

**FIG. 4.**
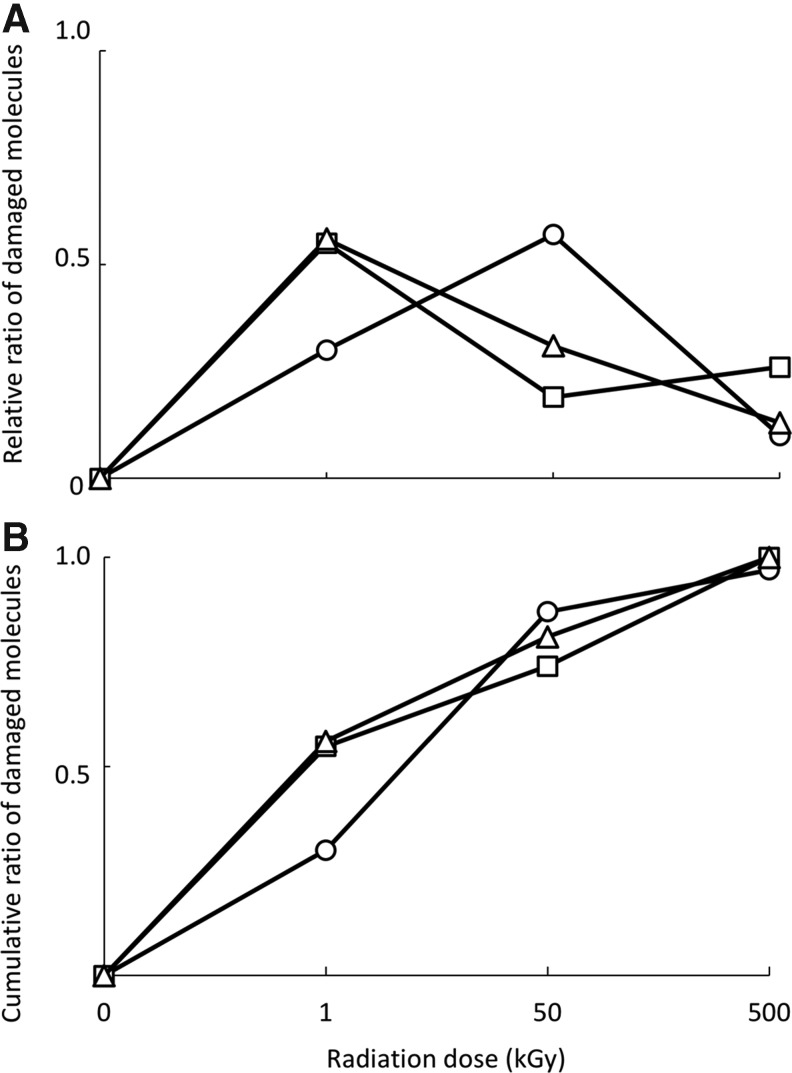
Dose–effect pattern of the molecular damage and cumulative effects of ionizing radiation. **(A)** Relative proportion of molecules newly affected with respect to the total, 31 for electron (squares), 30 for gamma (circles), and 16 for electron+gamma (triangles) radiations, which showed significant damage by Tamhane's T2 tests with respect to the nonirradiated control (0 kGy) after each radiation dose (see letters in Tables 3–5), that is, molecules affected at 1 kGy, affected at 50 kGy, and nonaffected at 1 kGy, and only affected at 500 kGy. The proportion of molecules affected by electron only or gamma radiation only showed statistical significant differences in all radiation doses (revealing by two-sample tests of proportions), whereas this number of affected molecules was not statistically significant when both radiations were applied in comparison with electron only and gamma only in all doses. Proportion of molecules irradiated with electron+gamma radiations showed a similar effect as that of the individual treatments as radiation dose increased although with a more similar pattern to that obtained with molecules irradiated only with electrons. **(B)** Cumulative damage (proportion of molecules statistically significant with respect to the nonirradiated control, 0 kGy); (see letters in Tables 2–4) with increasing radiation doses. Electron (squares), gamma (circles), and electron+gamma (triangles). See text for explanation and discussion.

After irradiation, the glass slides suffered a strong darkening with increasing radiation doses, until getting a deep brown color in the slides irradiated with 500 kGy (Fig. 1C). The temperature of the slides was <24°C during irradiation except for those irradiated with 50 and 500 kGy (Table 2), where it reached 45°C and 50°C for 1 s and as much as 10 s, respectively. After exposure to 1 kGy of electron radiation only, 55% (17/31) of the tested molecules lost a statistically significant immunoidentification signal with respect to the control, 71% (22/31) (from which 19%, 6/31, were not affected by 1 kGy) after being irradiated with 50 kGy, and 100% (31/31) (from which 26%, 8/31, were not damaged neither by 1 kGy nor 50 kGy) after 500 kGy (Table 3 and Fig. 4A). After 500 kGy of electron radiation, FtsZ (a bacterial division protein) and BFR (a bacterioferritin) peptides, with 16 and 17 amino acids, respectively, along with mellitic acid epitopes retained >75% of their nonirradiated control immunoidentification signal (Table 3). Unexpectedly, Tamhane's T2 test showed that, after 1 kGy of electron radiation, 3% (1/31, mellitic acid) of the compounds increased the immunoidentification signal (Table 3 and Fig. 3).

To discard or confirm any side effect of the 50°C reached during the 500 kGy electron radiation, microscope slides with the printed target molecules were heated at 50°C, 70°C, and 100°C for 15 min and then immunoassayed with the same antibodies as mentioned. We measured a reproducible loss in the immunoidentification signal in 37% of the target molecules after exposure to 50°C, whereas only 19% remain affected at 70°C, and, unexpectedly, none of them were affected after being incubated at 100°C (not shown).

### 3.3. Gamma radiation exposure

In the case of gamma radiation, after exposure to 1 kGy, only 30% (9/30) of the tested molecules lost a statistically significant fluorescence signal with respect to nonirradiated control. This percentage was increased to 87% (26/30) after 50 kGy and to 97% (29/30) after 500 kGy of gamma radiation. After irradiation with 50 kGy and 500 kGy, only 57% (17/30) and 10% (3/30) were newly affected molecules, that is, molecules affected by 50 kGy and not affected by 1 kGy, and molecules only affected by 500 kGy, respectively (Table 4 and Fig. 4A). Only the 15 amino acids ModA1 peptide conjugate (from a molybdenum transporter protein) did not show a statistically significant loss of immunoidentification signal after 500 kGy, retaining >80% of the nonirradiated control signal (Table 4). Tamhane's T2 test showed that after 1 kGy of gamma radiation, 17% (5/30 of all tested molecules) increased in immunoidentification signal (Table 4 and Fig. 3). These results indicate that at low doses, the exposure to electron radiation only was more harmful than exposure to gamma radiation only (Tables 3 and 4 and Figs. 3 and 4); but this was reversed at the highest radiation dose (500 kGy) (Tables 3 and 4 and Fig. 3).

### 3.4. Electron+gamma radiation exposure

Regarding the molecules exposed to electron+gamma radiations, 56% (9/16), 75% (12/16), and 100% (16/16) lost a statistically significant immunoidentification signal, with respect to the control, after 1, 50, and 500 kGy dose, respectively. After exposure at 50 and 500 kGy, 56% of molecules affected by 1 kGy decreased to 31% (5/16) and to 13% (2/16) of molecules newly affected, respectively (Table 5 and Fig. 4A). After 250 kGy of electrons and 250 kGy of gamma radiation, FtsZ and RRO peptides retained >75% of their control immunoidentification signal (Table 5). Tamhane's T2 test showed that after 1 kGy of electron+gamma radiations, 13% (2/16 of all tested molecules) increased in immunoidentification signal (Table 5 and Fig. 3).

Results of two-sample tests of proportions demonstrate that molecules exposed to electron+gamma radiations did not show statistically significant differences in the immunoidentification signal loss regarding the molecules irradiated with electrons only or gamma radiation only at all doses. Exposure to electron+gamma radiations had a similar effect as the separate treatments with increasing radiation doses, although with a more similar pattern to that obtained with molecules irradiated only with electrons (Fig. 4B).

### 3.5. Radiolysis rates

**FIG. 5.**
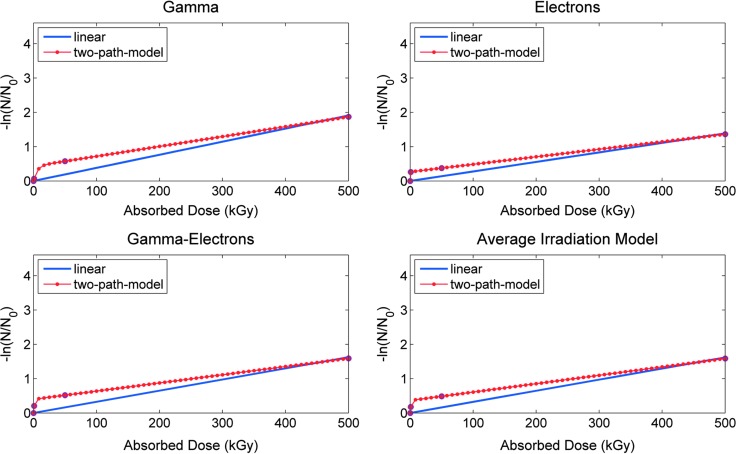
Comparison of linear and two pathways radiolysis models. Average surviving ratio (*N/N*_0_) of the molecules after irradiation with gamma, electron, gamma+electron, and the averaged irradiation among them, as indicated, at different irradiation doses (0, 1, 50, and 500 kGy). *N* is the fluorescence intensity of the immunoassay on the irradiated samples, and *N*_0_ the fluorescence intensity of the immunoassay on the nonirradiated control. Radiolysis rates were calculated as described in Section 2.7.

We determined the radiolysis rates from the immunoassay results as an indirect measure of damage of the target molecules after irradiation by the two models defined in Section 2.7. The linear model described in Equation 1 did not produce a good fit, mostly due to a clear decrease in the *N/N*_0_ ratio even for low radiation doses. This sharp decrease in the *N/N*_0_ ratio at lower doses was not observed with increasing radiation doses, and it seemed variable depending on the type of radiation. The destructive effect was more severe in cases where electrons were involved (electrons and gamma+electrons) (Fig. 3 and Tables 3–5). Although within a certain degree of uncertainty, mainly attributed to the limited number of radiation doses used, the two pathways model considers different contributions for low and high radiation doses that could to be more appropriate for dealing with complex molecules, even when simple epitopes are part of larger conjugates, whose response to radiation may be produced at multiple levels (Table 6 and Fig. 5). Considering the average values, gamma radiation seemed to be the most harmful radiation at epitope level, as we already showed for the highest dose (500 kGy), whereas electron radiation seemed to be the most harmful at low doses (Tables 3–5 and Figs. 3 and 5).

**Table 6.** Parameters for the Two Radiolysis Models Used in This Work

	*Linear model*		*Two pathways model*			
*Radiation*	k_r_	R^*2*^	A	k_*1*_	B	k_*2*_
Gamma	0.00382	0.94655	0.64941	0.00288	0.35059	0.20151
Electrons	0.00277	0.91969	0.76604	0.00219	0.23396	3.90680
Gamma + electrons	0.00325	0.92068	0.67133	0.00238	0.32867	0.82925
Average radiation	0.00324	0.93482	0.69491	0.00244	0.30509	0.73989

### 3.6. A case study for Mars

**FIG. 6.**
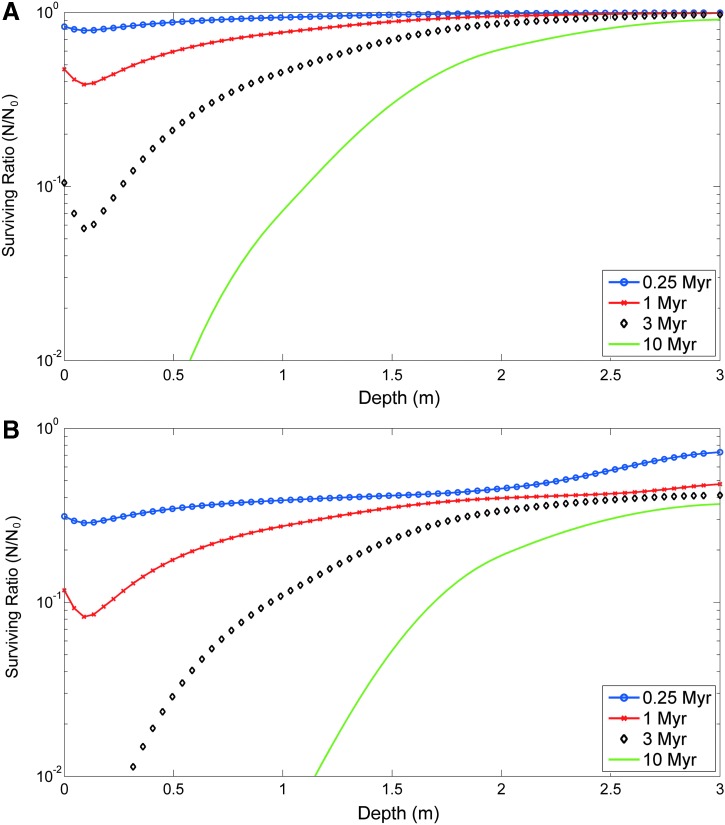
Depth-dependent surviving fraction (*N/N*_0_) of the irradiated polymeric compounds, simulated by the linear model **(A)** and the two pathways model **(B)**, after being exposed to 0.25 (green), 1 (blue), 3 (red), and 10 Myr (gray) to the ionizing radiation in the Martian subsurface at the MSL landing site. Target molecular biomarkers would still maintain immunoidentifiable structural features after 10 Myr radiation between 1 and 2 m depth (dotted vertical lines). Differential damage of the molecules under martian radiation may render altered products with different degrees of immunoidentification efficiencies along 10 Myr period. MSL, Mars Science Laboratory.

The two radiolysis models already described were applied to a particular case study on the martian surface after the radiation doses previously reported (Hassler *et al.*, 2014). From the average radiations, we adjusted the Hassler *et al.* (2014) results of the depth-dependent surviving fraction in the martian regolith to the linear and to the two pathways models (Fig. 6). Because the linear model (Eq. 1) did not present a good fit as a result of the sharp *N/N*_0_ ratio decrease for low radiation doses, it seemed to underestimate the radiation damage, particularly at high depths. Although the two pathways model (Eq. 2) might be overestimating the radiolysis effect, we considered it as a worst-case scenario. The results suggest survival ratios of 40%, 35%, 25%, and 5% for 0.25, 1, 3, and 10 Myr radiation equivalent, respectively, at 1.5 m depth (Fig. 6). As expected, the surviving ratio increased with the depth, so it was 45%, 40%, 35%, and 20% for 0.25, 1, 3, and 10 Myr, respectively, at 2 m depth. Clearly, the increment in the surviving ratio with the regolith depth is more relevant for long periods of time, for example, 10 Myr, than for short periods, for example, 0.25 Myr, in which it is even negligible. The local minimum between 0 and 0.5 m can be attributed to a higher radiative field intensity at these depths as reported by Hassler *et al.* (2014).

## 4. Discussion

### 4.1. Measuring the effects of ionization radiation on organic molecules by immunoassays

Because immunoassays mainly target small regions of larger molecules (*i.e.*, epitopes), much can be learnt from them regarding the effects of ionizing radiation on the chemistry and the structure of organic molecules, including biogenic molecules. There is extensive literature reporting that the radiation sensitivity is dependent on the size of the macromolecules (Kempner and Schlegel, 1979; Venter *et al.*, 1983; Peters *et al.*, 1984), and the fragmentation effect of radiation on biopolymers such as proteins (Miller *et al.*, 1998), DNA (Rydberg, 1996), or polysaccharides (Edwards *et al.*, 1977; Atrous *et al.*, 2015) has also been reported. Some experimental studies in the planetary exploration context showed a linear increase of the radiolysis constant with the molecular weight in small molecules such as amino acids (Kminek and Bada, 2006).

Our results show that, although the immunoidentification of very large complexes as spores, EPS and LPS were more severely affected, especially when they were irradiated with electrons, the extent of the immunoidentification loss with increasing radiation did not follow a clear pattern or an association with epitope complexity or the size in the rest of molecules (Fig. 3 and Table 3). The former observations were in agreement with the work of Pawlowski and Svenson (1999), which showed a loss in the immunoidentification of full length bacterial polysaccharides after depolymerization induced by electron radiation (from 12 to 200 kGy). In our study, the lack of a clear correlation between the size of the target molecules and the radiation effect on their immunoidentification may be explained by the fact that, except the target proteins, all the rest of the targets are in the form of hapten conjugate complex with another protein (BSA, KLH, or OVA). This means that, although the epitopes were small molecules (even amino acids), all the conjugates might behave as macromolecules.

The pattern of the damage produced by each radiation type indicated that electron radiation affected >50% of the molecules at 1 kGy, whereas gamma radiation damaged 30% (Tables 3 and 4 and Fig. 4). At 50 kGy, gamma radiation damage increased in >50% of the newly damaged molecules (*i.e.*, those affected at 50 kGy but not at 1 kGy), whereas electrons lowered their damage rate to 20% of newly affected molecules and increase slightly after 500 kGy. These differences in affecting new molecules indicate that gamma radiation needs higher doses than electron radiation for damaging more molecules, whereas electron radiation had reduced effect >50 kGy. Although penetrating capacity of electrons is much below the gamma radiation, it is always at the centimeter scale (Wang and Brynjolfsson, 1983). Considering that the thickness of the irradiated microarray spots is always <10 μm, this different behavior should not be related to the penetration capacity of both radiation forms, but mostly to the intrinsic physicochemical mechanisms operating for each radiation type (Wang and Brynjolfsson, 1983; Urbain, 1986). Alternatively, the results suggest that the damages to the epitopes due to electrons reach the saturation regime at lower doses than gamma rays. This may depend on the higher cross section of the electrons at 1 kGy with respect to gamma particles, but there may be other factors, such as the higher LET of electrons that may severely affect the molecules even at lower doses. The cumulative proportion of molecules with significant damage with respect to the nonirradiated control increased with gamma radiation, up to 87% at 50 kGy, whereas that of electron radiation was 71% (Fig. 4). However, the final cumulative damage after 500 kGy is slightly but significantly higher with electron radiation.

 When electron+gamma radiations were applied to the target compounds, the dose–effect pattern was similar to that of the individual treatments, although with a smoother slope and nearly linear pattern, but not an additive behavior. At high doses (mainly at 50 kGy), the proportion of molecules affected by irradiation with electron+gamma was between the individual radiations. This effect indicates that, although there might be some mechanistic differences in the mode of action of each type of radiation, as deduced by their different dose–effect patterns, it is the amount of radiation (dose) and not the type of radiation that is the main parameter conditioning the cumulative harmful effect on the molecules at high doses, as revealed in the two-way ANOVA. This is relevant for experimentation about the radiation effects on the organic/biological matter in the context of planetary exploration, where it is required to simulate high radiation doses and long periods of exposure.

Another significant finding in this study is that exposure to the smallest dose (1 kGy) of gamma radiation resulted in an increase in the immunoidentification of almost 20% of the molecules tested. Although the beneficial effect of low ionizing radiation doses on the stimulation of the immune system in animal models is well known (Shimura and Kojima, 2014), it is mainly attributed to the stimulation of antibody production and specific cell types by products released by damaged cells (Yang *et al.*, 2014). Hati *et al.* (1990) reported that the antiserum produced against gamma-irradiated viper venom had better neutralizing efficiency when the antiserum was produced with the venom irradiated at a lower dose. However, to the best of our knowledge, there are no reports in the literature showing a direct effect of gamma radiation on the epitope–antibody binding as that shown herein. All molecules within this group were proteins and peptides, and one possible explanation for this observation is that small levels of radiation may cause the partial unfolding of the protein tertiary structure, complete denaturation, or a slight conformational change (*e.g.*, by ionization), thereby exposing more linear epitopes to which antibodies can bind (Forsström *et al.*, 2015). This increase in immune detection was much more subdued in the case of electron radiation, which appeared to be more efficient at suppressing immunoidentification at low radiation doses.

Although less penetrating, the fact that of the energy we used was higher than the gamma radiation (10 MeV vs. 3 MeV, respectively) could explain its higher effect at low doses. Alternatively, electron radiation might be generally more harmful than gamma radiation due to higher interaction with matter (Wang and Brynjolfsson, 1983). The latter might be more realistic, because considering the thickness of the spotted compounds was around 10 μm, the differences between both types of radiation regarding the penetration effect may be considered negligible. Finally, although the molecules assayed were covalently bound onto the slides, we cannot rule out the possibility that radiation might have damaged their chemical link to the support. Considering that the molecules are theoretically attached to the slide through several covalent bounds, the contribution of this damage should be statistically much lower than the sum of all internal damages of the molecules.

The strong darkening effect on the microscope glass slides was in agreement with a similar effect reported by Allen *et al.* (1999), when a series of Mars analog rocks and minerals were subjected to sterilizing gamma radiation doses (300 kGy). The only effect detected was the darkening of quartz and halite as well as an increase in the albedo of carbonates. No measurable changes were detected in the isotopic or the chemical composition. Similarly, we did not find any correlation with the loss or gain of immunoidentification capabilities associated with slide darkening nor in this study nor in previous work about the effect of radiation on antibody performance (de Diego-Castilla *et al.*, 2011).

All the irradiation experiments were conducted under ambient temperature in the facility rooms, but during electron-only irradiation, temperature increased up to 50°C in the 500 kGy samples for 10 s. It is known that the sample temperature may affect the free radical formation rate (Lee *et al.*, 2001) mainly in liquid state and, therefore, the preservation of the activity of the molecules (Kempner *et al.*, 1986). However, considering that all our samples were in dry state, and we irradiated them in a narrow stretch of temperatures, the production of free radicals and their effect on the structure of the compounds are expected to be proportional to radiation dose but, mostly, negligible. After the finding of the dependence of radiation sensitivity with temperature on the activity of biological polymers described by Kempner *et al.* (1986), it is expected that the freezing temperatures of the martian surface would impair the free radical production and diffusion, contributing to slower the degradation of the organic matter.

Another effect of high temperatures may be the damage on the structure of the molecules by thermal denaturation. We previously reported that at least 50°C was not enough even to alter the functionality of printed antibodies on glass slides (de Diego-Castilla *et al.*, 2011). The results we have obtained after exposing several of the molecules used in this study at different temperatures (see Section 3) reflect the complex nature of the biorecognition events. In the affected molecules, conformational epitopes might be destroyed at 50°C, whereas as temperature increased to denaturing ranges (70°C and 100°C), new linear epitopes are formed and compensate for the initial loss of the conformational epitopes. Because the antibodies are polyclonal, a subset of antibody molecules might recognize different subsets of epitopes, some affected at 50°C and other created after temperature denaturation.

Whatever the case, there was no correlation between the loss of immunoidentification signal in the heating experiment and the loss of immunoidentification signal in the 500 kGy electron irradiation. For example, two of the molecules (BFR and FtsZ) that decrease 53% and 31%, respectively, with respect to the ambient temperature control, only lost 21% and 12.1%, respectively, in the irradiation experiment. In addition, the molecules (62.5%) that were not affected by temperature treatment showed a significant decrease in the detection signal after 500 kGy electron radiation. Therefore, we assumed that the effect of temperature of 50°C or slightly over it reached for only a few seconds during 500 kGy electron radiation (Table 2) was not significant enough to affect the structure of the printed compounds, and consequently its contribution to the loss of fluorescent signal was negligible.

### 4.2. Astrobiological implications for the search of biomarkers in planetary exploration

In this study, we worked on the assumption that the hypothetical life on other planetary bodies in the Solar System is based on the same universal principles of biochemistry (Pace, 2001). We think that finding biochemical compounds (from aromatic amino acids, nucleotides, peptides, complex sugars, and lipids) and the biopolymers they can form is an appropriate strategy to get evidence of life that is broadly similar to Earth life. The bioaffinity systems as the immunoassays are highly sensitive and allow detecting chemical structures and identify biochemical compounds. Therefore, we need to understand how these targets might be affected by ionizing radiation, one of the most harmful agents on the surface and near subsurface of planets with thin or without atmosphere. All the target molecules used fit in this objective, most of them because they are universal biochemicals while others are examples of complex chemical architectures that could be found on Mars (Benner *et al.*, 2000; Freissinet *et al.*, 2015).

Matthiä *et al.* (2017) reported the modeling of the complex radiation field on the surface of Mars from the radiation measurements by the NASA's MSL RAD instrument. There is no single facility that can simulate all types of radiations (protons, neutrons, HZE ions, gamma, electrons, muons, etc.) reaching the surface of Mars. Herein, our objective is to understand how accumulated ionizing radiation on target molecules/complexes may affect their immunoidentification. Owing to the experimental constraints in simulating planetary environment radiation scenarios (high-energetic particles, freezing temperatures, defined atmospheres, and availability of radiation facilities), gamma and electron radiations have been traditionally used to study the effect of ionizing radiation on biomolecules (Kminek and Bada, 2006). Both are abundant on the martian surface and, although gamma radiation is highly penetrating, the electrons are highly attenuated at micrometer scales and are widely used in sterilizing procedures (Yaman, 2001). With the corresponding limitations, the radiation doses utilized in our experiments were selected to represent the accumulated ionizing radiation equivalent to exposure times of 12,000 years, 0.6, and 6 Myr on the martian surface, based on the simulated results from real measurements (Hassler *et al.*, 2014). That is, we are considering the hypothetical scenario that all the radiation on Mars is gamma, electrons, or gamma+electrons, and the damage level produced by these types of radiation is then corrected to the martian levels published in the work of Hassler *et al.* (2014), taking into account the contributions of other types of radiation such as protons or HZE ions. This particular choice reflects the expected threshold of survival of complex organic molecules in the martian environment based on previous experiments (Dartnell *et al.*, 2012), and also the current reach (down to 1 m depth) of sampling systems on proposed robotic missions such as *IceBreaker* (McKay *et al.*, 2013).

To study the effect of ionizing radiation on the structure of organic molecules in a planetary relevant environment, it is required to control or simulate the temperature and the gas composition during the irradiation experiment (Kminek and Bada, 2006; Gerakines and Hudson, 2013). In our study, owing to the experimental and facility constrains, together with the large number of samples, we performed the irradiation under air and room temperature. We considered these conditions as a worst-case scenario where the radiation effect might have been increased by secondary events such as ozonolysis. Our focus was in understanding the effect of different types and doses of ionizing radiation on a large number and diverse molecular structures and, at the same time, in validating a high-throughput technique as protein microarray for a quick and efficient evaluation of the radiation damage.

Although we have to be cautious in extrapolating our data to a martian scenario (more realistic experimental setups are now underway to approach Mars-relevant conditions), and considering the radiation effect alone, our results under this worst-case scenario allow for estimation of the target survival ratio *N/N*_0_ as a function of the martian regolith depth, for four illustrative exposure times (0.25, 1, 3, and 10 Myr) at the MSL landing site (Fig. 6). The dose rates have been obtained from the work of Hassler *et al.* (2014), and the *N/N*_0_ ratio was computed by the previously considered models, described by means of Equations 1 and 2, using the models for an average radiation stated in Table 6.

After selecting the two pathways model as the “worst”-case scenario, our results reveal a surviving ratio of ∼20% in molecules located in the martian regolith at 2 m depth and exposed at a dose of ionizing radiation equivalent to 10 Myr (Fig. 6). Based on these data, for example, the ExoMars 2020 rover, which is expected to drill in the martian regolith to 2 m depth, should be able to find similar organic molecules/compounds in the martian regolith if the hypothetical targets were exposed to radiation for time periods <10 Myr.

These results are based on a constant radiation field reaching the surface within a period of 10 Myr, and for regolith features similar to the MSL landing site. Other scenarios should be considered for future irradiation experiments that include different oxidative conditions of the martian regolith, the presence of water ice, or different cryogenic temperatures. Although using equivalent dose rates allows a rough normalization of the biological damage, other types of ionizing radiation such as protons and neutrons have to be considered to further our comprehension of the radiation effect on the aforementioned molecules and compounds.

Altogether, our results suggest that immunodetection of organic compounds of biological origin within the top 2 m of the martian regolith would be possible for exposure times <10 Myr, but it would become increasingly difficult in longer timescales (Fig. 6). This timescale is comparable with the loss of prominent spectral features from biomarkers determined with Raman spectroscopy (Dartnell *et al.*, 2012), and point to an absolute limit to biomarker recognition as a function of radiation exposure. Arguably, this absolute limit does not apply to all biomarkers. Biomolecules protected by minerals (Hassink, 1997) or highly resistant biomarkers such as lipids and their derivative hydrocarbons (Martins, 2011) might be more resistant to ionizing radiation.

Our experiments were carried out under a terrestrial environment (room temperature, air, and atmospheric pressure) and, therefore, additional experiments are needed to simulate other planetary environments as Mars or Europa. Still, our results are relevant in the context of astrobiological missions for searching for molecular evidence of life, such as the *IceBreaker* mission concept to drill into the martian permafrost in the northern terrains (McKay *et al.*, 2013). Recent Mars habitable environments might have been created in the past 10 Myr as a consequence of recurrent insolation periods on the martian permafrost (Laskar *et al.*, 2004; Richardson and Mischna, 2005).

Although the different radiation types and doses used in this study are relevant, we understand that further improvement of the experimental setup is needed. For example, cryogenic temperatures and the presence of water ice matrix. Gerakines *et al.* (2012) and Gerakines and Hudson (2013) reported an increase in the destruction rate of gamma-irradiated glycine with decreasing temperatures from 140K to 15K, as well as a shielding effect of water ice. The fact that they reported also a higher degradation rate at 280K together with some effects related to the concentration of the molecule illustrates how complex is the simulation of the effect of ionizing radiation on planetary environments over molecules even as simple as glycine. In our case, we have multiple macromolecules with high diverse structures that might be affected in different ways. We are currently performing experiments with a selected set of 15 amino acid peptides, where we have identified 4–5 amino acids epitopes, to approach the radiation effect under more realistic Mars and Europa environments, where low temperatures and an ice matrix will be considered.

Alternatively, more realistic simulation scenarios can be achieved by long-time exposure of similar molecules as we have used herein in the International Space Station (ISS) facilities to address the effect of multiple radiation types, energies, and dosages. Examples of such studies have been reported (Cockell *et al.*, 2011; Horneck *et al.*, 2012; Tepfer and Leach, 2017) where phototrophic biofilms, *B. subtilis* spores, or plant seeds have been exposed to space conditions in the ISS for >600 days. However, simulating radiation doses equivalent to millions of years of accumulative radiation on a planetary-relevant environment is extremely complicated, mainly due to the low ionizing radiation rates at the ISS and the large amount of time of use that would be required to simulate planetary timescales.

## 5. Conclusions

We have used microarray-based fluorescent immunoassays to study the harmful effect of ionizing radiation on organic and biological molecules. Although this study does not show which detailed chemical modifications occurred within the molecules after irradiation, the loss of fluorescence after the immunoassay with increasing radiation doses is an indicator of any structural or chemical alteration of the antigen and/or the epitopes directly induced by ionizing radiation. This approach can be applied to the study of structural alterations induced by radiation in biochemistry, medical fields, or under highly irradiated planetary surfaces. Further experiments with techniques used in the study of protein structure, for example, X-ray crystallography of antibody–epitope complexes before and after irradiation, are necessary to understand the fine chemical modifications on epitopes in target molecules. However, these studies are beyond the scope of this work.

Our results indicate that, for gamma rays and electrons under the experimental conditions described herein, it is the dose and not the radiation type that is the most significant factor in the loss of immunoreactivity and, therefore, in the molecular damage at high doses. The electron-only radiation was more effective than gamma-only radiation in suppressing the immunoidentification at low radiation doses, whereas this was reversed at the highest dose (500 kGy). It is also remarkable and deserves further investigation that exposure to the smallest dose (1 kGy) of gamma radiation resulted in an increase in the immunoidentification signal of ∼20% of the molecules tested, mostly peptides.

Finally, our results suggest that high ionizing radiation doses equivalent to 10 Myr on the martian surface do not destroy completely the structural information of biological polymers. Such structures can be identical or highly similar to the epitopes initially recognized by a set of antibodies produced to bind them. Any factor or component that might contribute to protect the organic matter to the effect of ionizing radiation would increase their half-life. Therefore, biochip-based solutions (Parro *et al.*, 2011b; McKay *et al.*, 2013) or other bioaffinity-based system aimed to detect complex structures may be suitable for searching for similar molecular structures in short timescale radiation-exposed samples in planetary exploration.

## Abbreviations Used

BSA: bovine serum albumin
EPSs: exopolymeric substances
GCRs: galactic cosmic rays
HZE: high atomic number and energy
IgE: immunoglobulin E
ISS: International Space Station
KLH: keyhole limpet hemocyanin
LET: linear energy transfer
LPS: lipopolysaccharide
MSL: Mars Science Laboratory
Myr: Million years
OVA: ovoalbumin
SEPs: solar energetic particles
